# A human *in vitro* platform for the evaluation of pharmacology strategies in cardiac ischemia

**DOI:** 10.1063/1.5089237

**Published:** 2019-08-13

**Authors:** Carlota Oleaga, Golareh Jalilvand, Gregg Legters, Candace Martin, Gail Ekman, Christopher W. McAleer, Christopher J. Long, James J. Hickman

**Affiliations:** 1 NanoScience Technology Center, University of Central Florida, 12424 Research Parkway Suite 400, Orlando, Florida 32826, USA; 2 Hesperos, Inc., 3259 Progress Dr, Room 158, Orlando, Florida 32826, USA

## Abstract

Cardiac ischemic events increase the risk for arrhythmia, heart attack, heart failure, and death and are the leading mortality condition globally. Reperfusion therapy is the first line of treatment for this condition, and although it significantly reduces mortality, cardiac ischemia remains a significant threat. New therapeutic strategies are under investigation to improve the ischemia survival rate; however, the current preclinical models to validate these fail to predict the human outcome. We report the development of a functional human cardiac *in vitro* system for the study of conduction velocity under ischemic conditions. The system is a bioMEMs platform formed by human iPSC derived cardiomyocytes patterned on microelectrode arrays and maintained in serum-free conditions. Electrical activity changes of conduction velocity, beat frequency, and QT interval (the QT-interval measures the period from onset of depolarization to the completion of repolarization) or action potential length can be evaluated over time and under the stress of ischemia. The optimized protocol induces >80% reduction in conduction velocity, after a 4 h depletion period, and a partial recovery after 72 h of oxygen and nutrient reintroduction. The sensitivity of the platform for pharmacological interventions was challenged with a gap junction modulator (ZP1609), known to prevent or delay the depression of conduction velocity induced by ischemic metabolic stress. ZP1609 significantly improved the drastic drop in conduction velocity and enabled a greater recovery. This model represents a new preclinical platform for studying cardiac ischemia with human cells, which does not rely on biomarker analysis and has the potential for screening novel cardioprotective drugs with readouts that are closer to the measured clinical parameters.

## INTRODUCTION

Ischemic heart disease can occur as a consequence of a wide range of cardiovascular conditions, influenced by genetic background and lifestyle, and is currently the leading cause of death from noncommunicable diseases.^1^ During cardiac ischemia, the blood supply to the heart is partially or completely blocked, inducing damage in the affected area. The lack of oxygen induces a metabolic shift from oxidative phosphorylation to anaerobic glycolysis, which limits the cellular energy source to only glucose and amino acids until exhaustion, instead of fatty acids. The shortage of blood flow to the ischemic region induces a buildup of waste products in the extracellular space, decreasing the pH.^2^ Alteration in the ion homeostasis promotes changes in the excitation-contraction apparatus of the cardiomyocytes, such as arrhythmias.^3^ The electrical propagation in the cardiomyocytes will also be affected due to losses in cell-cell connections, affecting the conduction velocity of the tissue.^4^

A cardiac ischemic event is diagnosed by observing changes in the electrocardiogram wave form, where an increase in the ST-segment is the most defined sign for myocardial infarction (ST-segment is a period in the electrocardiogram reading between the ventricular depolarization and the repolarization that is electrically neutral).^5^ Altered levels of troponin and creatine kinase in the bloodstream are also indicative of cardiac damage.^5,6^ Reperfusion is the first line of therapy in cardiac ischemia, and it can be performed by mechanical (percutaneous coronary intervention) or pharmacological (fibrinolytic therapy) intervention. These protocols have improved the survival of patients to ≥50%; yet, the time of treatment plays a critical role in treatment success.^5–8^ Reperfusion protocols, although effective in reducing damage in the affected area during the initial stages of the event, paradoxically can also induce a later damage to the tissue, called reperfusion injury as sudden blood flow recovery in the ischemic area can be detrimental for the heart.^7^ Strategies that may help reduce the shock of the reperfusion process are also under study by targeting vasodilation,^2,7,9^ mitochondrial function,^2,9–12^ cellular metabolism,^2,10,11,13–15^ ion imbalance,^15,16^ and cell-cell interactions.^4,17^ Although aspects of these therapeutic strategies hold promise, many have failed to demonstrate efficacy during preclinical testing. This poor success rate is raising questions concerning the validity of current preclinical models for studying ischemia.^4,7,17,18^

The current preclinical models approved by the International Council for Harmonisation (ICH) guidelines include small (mice, rat, rabbits, and guinea pig) and large (cats, dogs, pigs, and primates) animals as *ex vivo* and *in vivo* models. For animal models, the induction of ischemia is initiated by the occlusion of the coronary artery (the time of occlusion is specific for each animal model) and a later reperfusion. A significant difference in ischemic sensitivity between species hinders the scaling to humans.^7,18^ The scientific field is working to develop new *in vitro* models that will improve the understanding of ischemic physiopathology and to more accurately predict the activity of cardioprotective drugs during ischemic processes in humans. Some of these *in vitro* approaches are focused on cellular viability,^19–22^ molecular homeostasis,^23–26^ and functional changes^21–28^ upon the induction of an ischemic stress. However, only a small number of them use human cells in their models,^20–23,25,29^ and none combine them with functional determinations such as cardiac electrical conduction.

We report a functional human *in vitro* system able to measure the clinically relevant cardiac electrical activity functions [beat frequency, conduction velocity, and QT-interval (the QT-interval measures the period from onset of depolarization to the completion of repolarization)]^30,31^ from iPSC derived cardiomyocytes under ischemia stress for the evaluation of novel therapies. The sensitivity of the platform was validated with a gap junction modulator, ZP1609, known to protect cardiomyocytes from a reduction in conduction velocity under ischemic conditions.^32^ Upon an ischemic stress, the system had a faster recovery when treated with the gap junction modulator as compared to the control. These results successfully demonstrated the potential of the platform that measures clinically relevant parameters for testing molecules that protect or recover cardiac electrical properties affected by ischemic stress.

## RESULTS

### Human iPSC derived cardiomyocytes retain their electrical activity in serum-free medium for one month

**FIG. 1.**
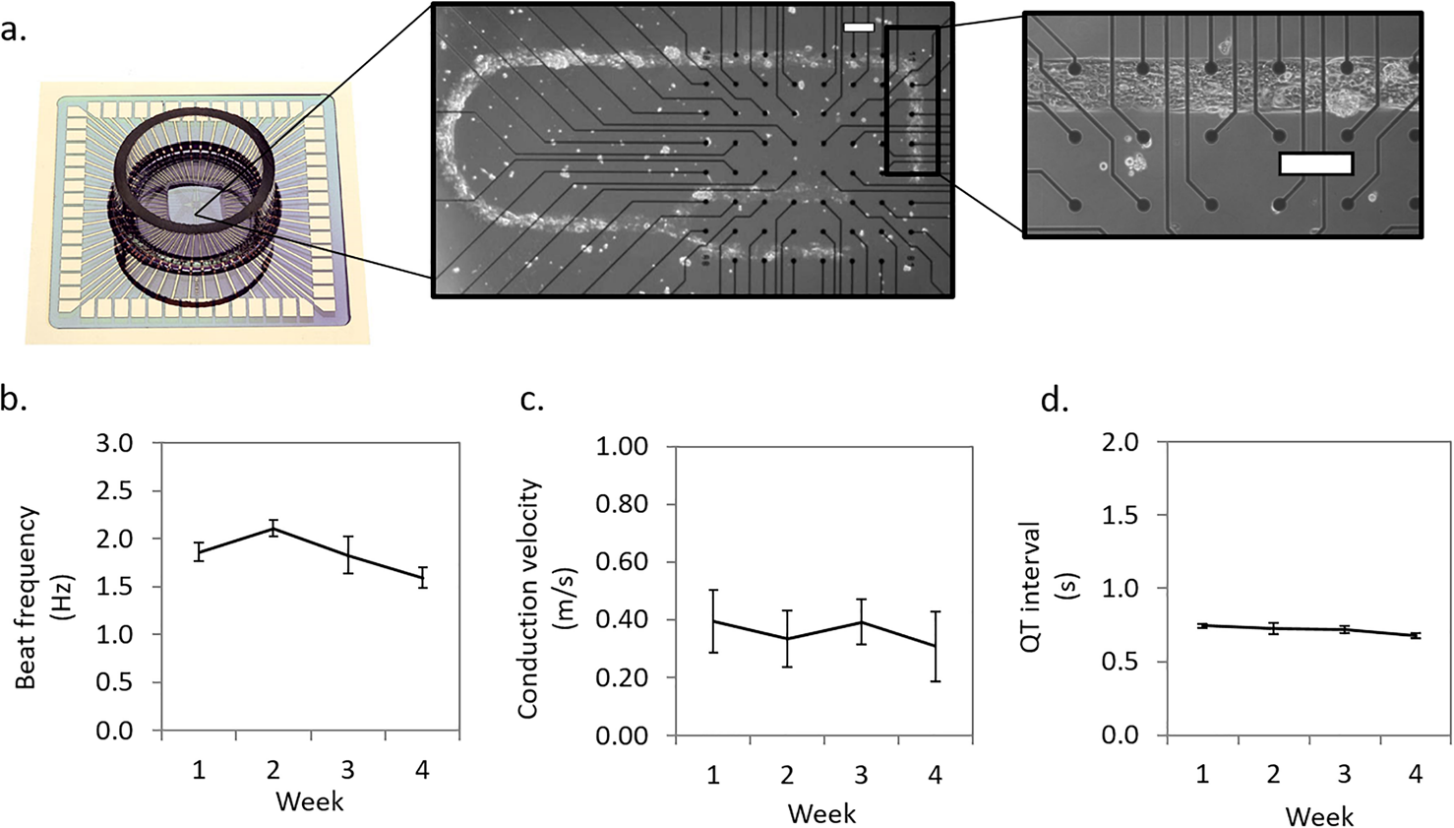
Characterization of human iPSC derived cardiomyocyte electrical activity in serum-free medium for 28 days. Representative morphology images are shown for patterned human cardiomyocytes on an MEA chip (200 *μ*m scale) (a). The cardiac electrical function was measured throughout 28 days and plotted as the mean ± SE (n ≥ 3) of spontaneous beat frequency (b), conduction velocity (c), and QT interval (d). A one-way ANOVA was performed to study the effects of the culture time on the different cardiac functional parameters; beat frequency (p = 0.06), conduction velocity (p = 0.9), and QT-interval (p = 0.15).

Human cardiomyocytes were cultured on multielectrode array (MEA) chips under serum-free conditions for up to four weeks. Cardiomyocytes were patterned on MEA electrodes as previously described^32^ to establish an organized cellular path of >3 mm long and 150 *μ*m width to enable conduction velocity studies under controlled cellular geometries [Fig. 1(a)]. In the serum-free media formulation (HSL2), the cardiac electrical functions [Fig. 1; beat frequency (b), conduction velocity (c), and QT-interval (d)] remained stable for over a month (all p > 0.05).

### Cardiac viability and phenotype are maintained after controlled ZP123 and ZP1609 incubation

**FIG. 2.**
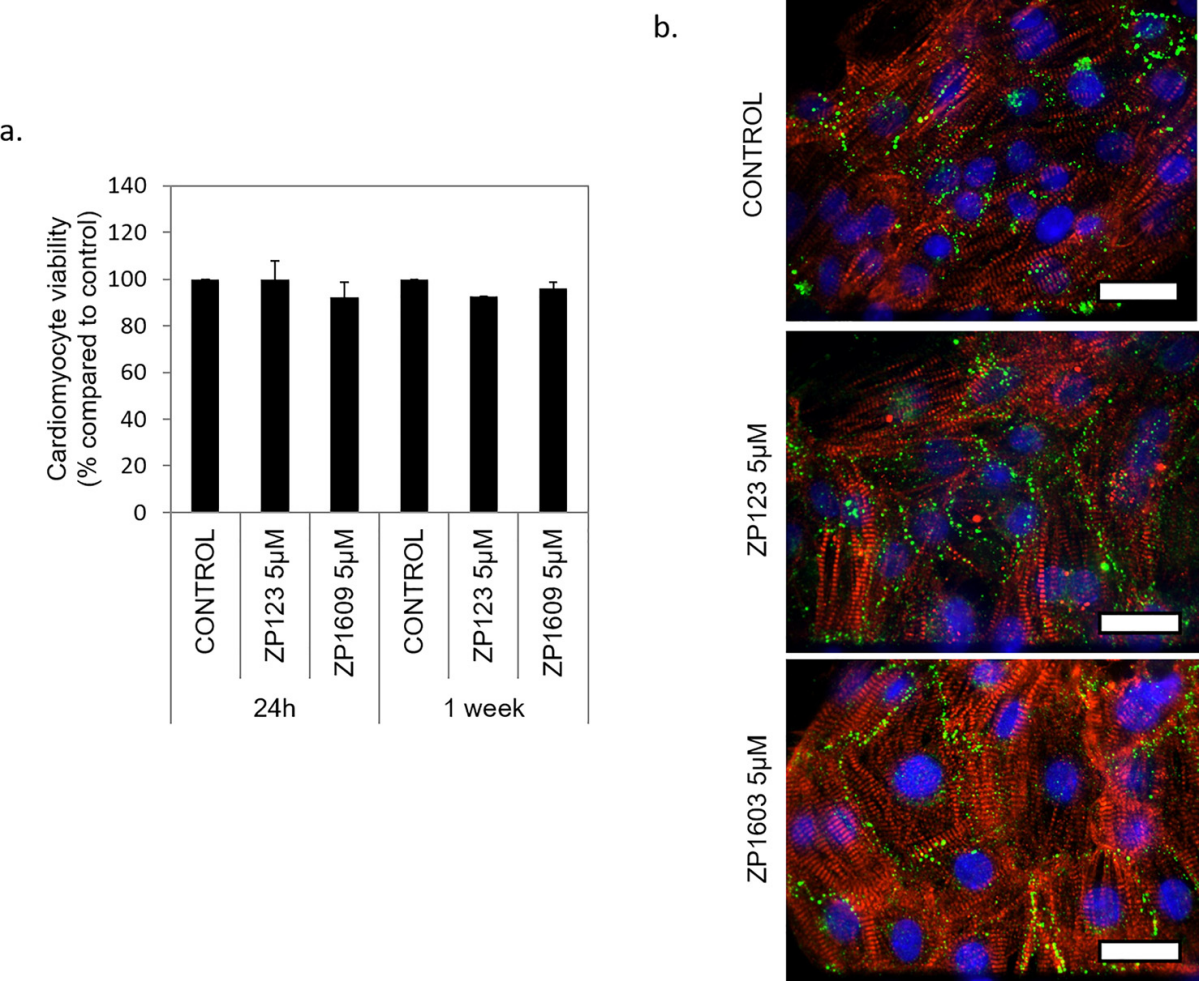
ZP123 and ZP1609 peptide effects on human iPSC derived cardiomyocytes. Nonpatterned cardiomyocyte (7 days *in vitro* or DIV) viabilities after incubation with ZP123 (5 *μ*M) or ZP1609 (5 *μ*M) peptides, for 1 and 7 days under control conditions, were plotted as the percentage of change to the control [mean ± SE (n ≥ 3)] (a). Representative immunocytochemistry images of troponin (red), connexin-43 (green), and nuclei (blue) stained at 7 DIV after 24 h of incubation with ZP123 (5 *μ*M) or ZP1609 (5 *μ*M) peptides (25 *μ*m scale) (b).

ZP123 and ZP1609 are two peptides developed to prevent or improve the damage induced in cardiac tissue after an ischemic event.^17,30,31,33^ They both have demonstrated the ability to protect cardiomyocytes from the detrimental effects of ischemia in cardiomyocyte electrical conduction^32,34,35^ in animal models. We evaluated the toxicity of these compounds on nonpatterned cardiomyocytes under control conditions, and human iPSC derived cardiomyocyte viability was not affected after 24 h or one-week treatment with either peptide at 5 *μ*M in HSL2 medium, indicating that the molecules may be safe for long-term treatment [Fig. 2(a)]. Under the same conditions, we also evaluated the expression and location of connexin-43, the main connexin molecule in the ventricle and atria of the heart,^4,33^ and troponin, a key protein involved in regulating the actin-myosin cross-bridge in the iPSC derived cardiomyocytes.^36^ After 24 h of incubation with these gap junction modulators, no visual changes appear to be altered by the compounds under control conditions [Fig. 2(b)].

### *In vitro* ischemia protocol optimization

**FIG. 3.**
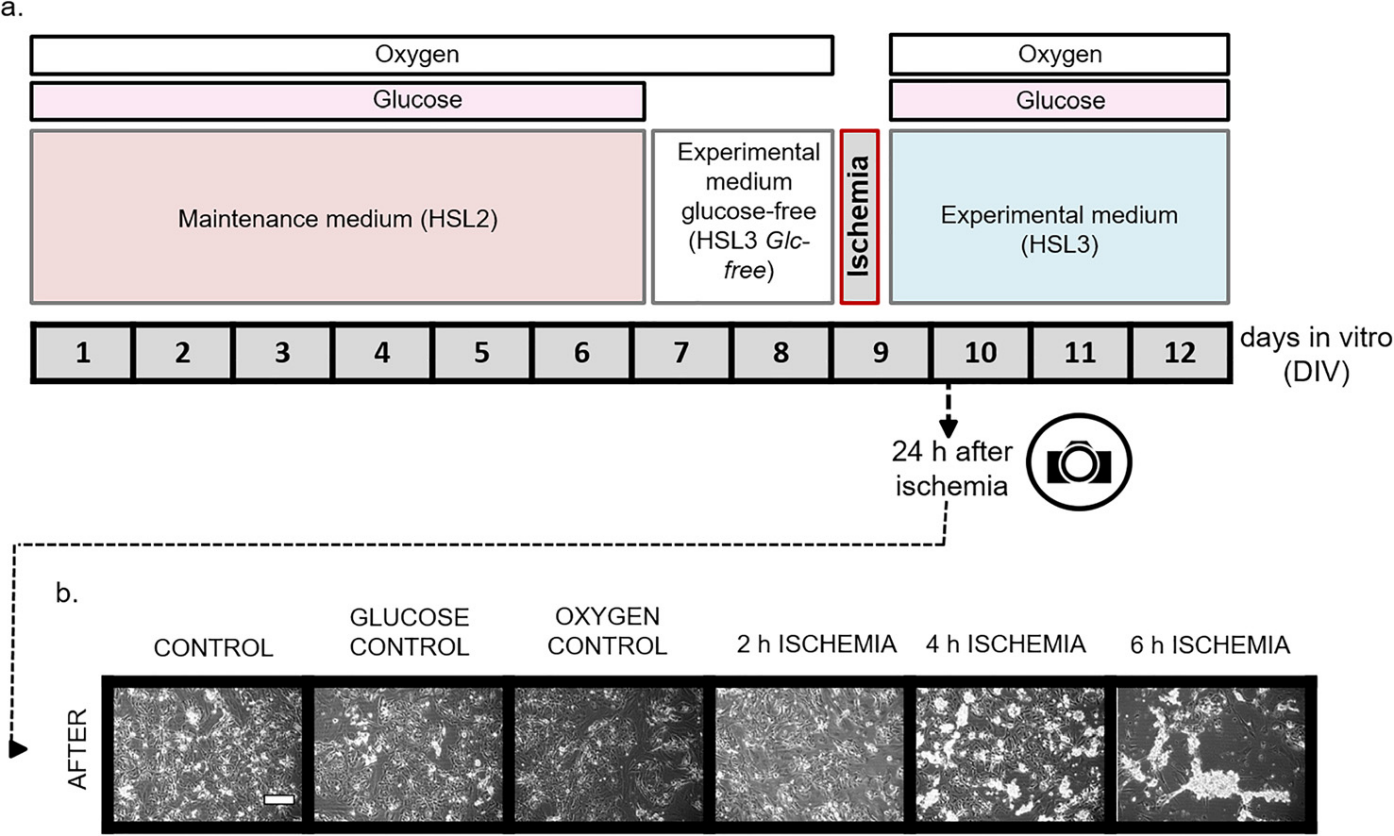
*In vitro* ischemia protocol and effect on human iPSC derived cardiomyocyte morphology. Schematic of the ischemia experimental protocol and timeline (a). Representative morphology images of cardiomyocytes under different conditions (Control: experimental medium and normoxia; Glucose control: experimental glucose-free medium and normoxia; Oxygen control: experimental medium and 4 h hypoxia; and 2–4–6 h ischemia: experimental glucose-free medium and 2–4–6 h hypoxia) at 10 DIV after the ischemia protocol (200 *μ*m scale) (b).

**FIG. 4.**
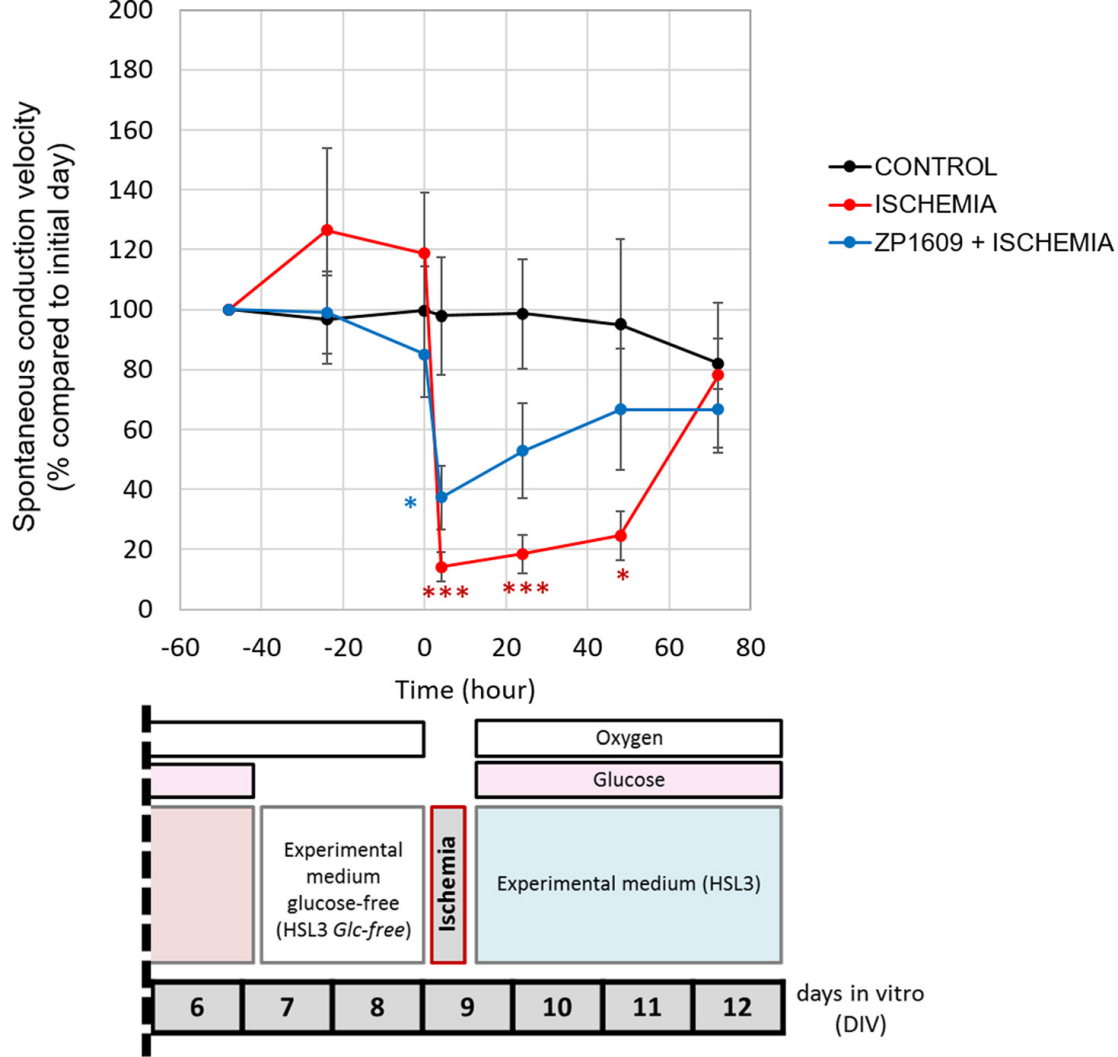
Ischemia effects on *in vitro* cardiomyocyte spontaneous conduction velocity. Spontaneous conduction velocity of patterned cardiomyocytes plotted as a percentage of change [mean ± SE (n ≥ 3)] compared to the initial measurement (at 7 DIV) of control (black) or ischemia conditions with (blue) or without (red) ZP1609 (5 *μ*M) preincubation (^*^p < 0.05; ^***^p < 0.001).

An *in vitro* ischemia protocol was developed to study changes in the electrical conduction properties of patterned cardiomyocytes. The protocol [Fig. 3(a)] was developed to disrupt the electrical function of cardiomyocytes with the possibility to recover after reoxygenation without the loss of cellular viability. Similar to what others have reported,^20,36,37^ cardiomyocytes required glucose depletion in the culture medium (amino acid and fatty acid sources were maintained) 48 h before oxygen depletion in order to exert measurable conduction velocity reductions. In brief, cardiomyocytes were plated, nonpatterned, and remained for 7 DIV (days in vitro) in the maintenance medium (HSL2). After this time period, the medium formulation was replaced to introduce the ischemic experimental condition. Experimental medium (HSL3) was introduced for both the control and oxygen control conditions, and the experimental glucose-free medium was introduced for the ischemia and the glucose control conditions. At 9 DIV, oxygen depletion was induced with the hypoxia chamber maintaining oxygen at a minimal level (0.1%–0.25%) during 2, 4, or 6 h. After the stress, oxygen and glucose were reintroduced and cultures were kept for three more days in order to investigate recovery. Morphology pictures 24 h after the ischemia period indicated that 6 h of ischemia was too detrimental for the tissue to correctly measure conduction velocity [Fig. 3(b)] even though the cardiomyocytes were still actively contracting (supplementary material 1). A period of 4 h of hypoxia was selected to enable the measurement of conduction changes in cardiomyocytes with the possibility of partial recovery. Cardiac spontaneous conduction velocity was reduced more than 80% after 4 h of ischemia and remained significantly low until 72 h after the applied stress [Fig. 4, supplementary material 3(b), and 4(a)]. This term of partial recovery was chosen for further experiments because spontaneous conduction velocity recovered after 72 h [Fig. 4 and supplementary material 3(a)]; however, stimulated conduction velocity failed [supplementary material 3(a) and 4(b)]. Although there was recovery of partial functions, cells appeared to be inexorably damaged (supplementary material 2). Controls to monitor conduction velocity alterations due to only glucose removal or only oxygen depletion or to the maintenance medium were also investigated (all p > 0.05) (supplementary material 3(b) and 4).

ZP1609 ameliorated the conduction reduction exerted by the ischemic event in cardiomyocytes *in vitro*. The system was challenged to detect changes in conduction velocity after the pretreatment of the cardiomyocytes with the gap junction modulator ZP1609. ZP123 was not further evaluated in this study since ZP1609 represented the next generation of the compound.^33,35,38^ At 7 DIV, the patterned cardiomyocytes on the MEAs with serum-free medium were used to study conduction alterations during ischemic conditions. Conduction velocity was monitored throughout five days, and changes over time were plotted as the percentage of change compared to the initial day of measurement (48 h) before inducing ischemia (time −48 h, 7 DIV). After the initial recording, experimental glucose-free conditions were introduced (time −48 h to 0 h) with or without the gap junction modulator. Oxygen depletion was introduced (time 0 h, 9 DIV) for 4 h to induce ischemia. After that, oxygen and glucose were reestablished and the function was monitored for the following 72 h, until 12 DIV (Fig. 4 and supplementary material 3 and 4). Cardiomyocytes under 4 h of ischemia required 72 h to recover their conduction velocity baseline; however, the pretreatment of cardiomyocytes with the peptide ZP1609 (5 *μ*M) significantly accelerated the recovery time to 24 h under spontaneous or 48 h under stimulated electrical conditions [Fig. 4 and supplementary material 3(a) and 4]. Although the ZP1609 peptide effectively protected cardiomyocytes from the detrimental effect of ischemia by allowing a faster recovery of the initial conduction velocity, the peptide showed no effects on the conduction function of control conditions [supplementary material 3(b) and 4].

## DISCUSSION

Advances to prevent and treat cardiac ischemia are necessary to improve survivability of this often fatal condition. Investigations utilizing new strategies to protect the cardiac tissue from the stress of ischemia by pre- and postconditioning the tissue,^7^ minimizing the energetic cost,^7,13^ preserving the connectivity,^4,13,17^ and quenching the oxidative stress^4,10,17^ are ongoing. Current preclinical models, especially animal models used to evaluate the efficacy of these new strategies, fail to accurately predict their outcome in humans;^7,10,18^ for this reason, better research platforms are necessary to evaluate the efficacy of these pharmacological compounds. We have developed a human *in vitro* platform that can measure cardiac conduction alterations induced by ischemic stress, and we have validated it for the study of pharmacological strategies that target the electrical function of cardiomyocytes. Our group has established a defined, serum-free system for measuring the electrical activity of primary rat cells^7,18,30^ and of human cardiomyocytes derived from both embryonic stem cells and induced pluripotent stem cells.^31,39,40^ Here, we have demonstrated that the *in vitro* system preserves the cardiac function for one month utilizing iPSC derived cardiomyocytes. The same system can be coupled to an ischemia protocol adapted from Refs. 22, 23, 26, 28, 34, and 41 to study *in vitro* electrical parameter changes that occur *in vivo* during ischemia.^4,5,42^ Under this protocol, cardiac conduction velocity is reduced, almost completely (>80% reduction) after a 4 h ischemia event, and only recovers back to its original velocity 72 h after the stress. Although there is a recovery in the spontaneous conduction properties, the recovery is not completed since under stimulated conditions, the cardiomyocytes fail to pace. This bioengineered *in vitro* system enables the measurement of the essential electrical functions of the heart,^4,5,30,31,42^ and when combined with the ischemic protocol, it allows us to study how an ischemic stress affects the cardiac function under defined conditions. We did monitor other electrical parameters such as the beat frequency, QT-interval, and field potential amplitude under spontaneous or stimulated conditions; however, no significant changes were found other than in conduction velocity (data not included). As a new *in vitro* system, it offers the possibility to screen for novel compounds that may exert cardioprotective effects on the electrical function of the heart under ischemia.

During ischemia, cell-cell communication is disrupted by the decoupling of gap junction connections. Gap junction modulators are a group of therapeutics known to play a protective role in those connections.^4,17^ ZP123 and ZP1609 peptides have been investigated for the indication of cardiac ischemia due to their antiarrhythmic and gap junction protective effects.^17,32,34,35,43^ Antiarrhythmic peptides, such as these, have been demonstrated to preserve the phosphorylation of connexin-43, inducing the stability of the gap junction coupling. By maintaining the cell-cell coupling, these peptides reduce the depression of conduction velocity induced by the metabolic stress of an ischemic event.^17,35,44^ Under control conditions (normoxia and glucose), the exposure to these peptides for 1–7 days at the concentration of 5 *μ*M had no effect on the viability of the cardiomyocytes. In addition, ZP1609 had no effect on the conduction properties of cardiomyocytes that were maintained under control conditions. Previous studies have demonstrated that these antiarrhythmic peptides do not exert changes in the conduction velocity of cardiomyocytes under physiological conditions.^34^ Overall, these peptides were safe for cardiomyocyte treatment, as expected from *in vivo* studies in animals and clinical trials previously run in humans.^33^ However, when ischemia was introduced (hypoxia and no glucose), ZP1609 significantly protected cardiomyocytes from the stress since it allowed the recovery of spontaneous electrical conduction two days before the control. Under stimulation, human iPSC derived cardiomyocytes that were preincubated with ZP1609 and then subjected to ischemic conditions also had a faster recovery compared to the control.

The presented cardiac *in vitro* system is a valid model for the study of ischemia physiopathology using electrical conductance of patterned human cardiomyocytes and has the potential to be used for novel therapeutic screening. The next generation of the model could incorporate a contractile force functional unit by applying cantilever technology previously developed by our group,^31,33^ to complement the study with changes in contractile force induced by ischemia^31,34,35,45^ and to enable the study of therapeutic groups targeting the contractile machinery of the heart under ischemic conditions. In addition, this technology could be translated to a multiorgan system that allows cross talk with other organs,^34,35,39,40,45,46^ as well as monitor organ functions for long-term periods.^47^

## CONCLUSIONS

A novel *in vitro* platform has been developed to study conduction velocity changes under human cardiac ischemic conditions. The platform retains human iPSC derived patterned cardiomyocyte electrical activity under serum-free conditions for 28 days. Drastic changes in cardiac conduction velocity occur upon ischemic stress. The gap junction modulator (ZP1609) validated the sensitivity of the ischemic *in vitro* platform to detect differences in recovery or protection to the disrupted conduction. This model represents a new functional preclinical platform for studying cardiac ischemia *in vitro* with human cells that can be used for screening of novel cardioprotective drugs.

## METHODS

The procedures performed in this study involving animals were in accordance with the ethical standards of the University of Central Florida IACUC Committee, titled “Realistic *in vitro* mimics of mammals for toxicity testing” Number 18-33.

### MEA chips

Multielectrode arrays (MEAs) (60MEA200/30iR-Ti, Multichannel-Systems, Germany) were used for extracellular recording experiments. Surface modifications of the MEA chips to pattern cardiomyocytes and cell plating protocols were previously described in Refs. 30, 31, 39, and 46.

### Cell culture

Cryopreserved human iPSC derived cardiomyocytes (current product number is R1007, Cellular Dynamic International, CDI) were thawed according to the manufacturer's instructions. Surfaces (18 mm diameter glass coverslips and patterned MEA chips) were sterilized with absolute ethanol. Next, a 10 *μ*g/ml fibronectin solution [in 1 × PBS (Phosphate-buffered saline)] was incubated for 30 min at 37 °C on the surfaces and rinsed three times with 1 × PBS. Cardiomyocytes were plated on glass coverslips (400 cells/mm^2^) and MEA chips (1000 cells/mm^2^) with maintenance medium (HSL2)^30,31,39^ and cultured with a half media change every two days until experiment initiation. Cell morphology was studied using an inverted phase contrast microscope (Carl Zeiss, Axiovert 200) using a 2.5 and a 10× objective. Images were collected using Axio Cam and AxioVision AC software.

### Experimental media

Experimental medium (HSL3) was adapted from Refs. 30, 31, 39, and 48 by adjusting the osmolarity to 300 mOsm/Kg with sodium chloride. The experimental “glucose-free” medium (HSL3 “Glc-free”) was formulated based on the experimental medium; briefly, Neurobasal base medium (Thermo Fischer Scientific, 21103049) was substituted by Neurobasal A (glucose and pyruvate free) (Thermo Fischer Scientific, A2477501) and supplemented with 0.22 mM sodium pyruvate. Osmolarity was equally adjusted with sodium chloride to achieve 300 mOsm/Kg.

### Extracellular electrical recordings

Extracellular recordings of the electrical activity of patterned human iPSC derived cardiomyocytes on commercially available MEA chips (Multichannel-Systems, containing 60 electrodes of diameter 30–60 *μ*m with a 200 *μ*m pitch) were acquired as previously described by our group.^30,31^ In brief, a cell-adherent foreground (fibronectin) surrounded by a cell-repulsive background [Polyethylene glycol (PEG)-silane] was created on the MEA surface to induce a cell patterning, by using surface modification and photolithography techniques. Cells were patterned in a linear shape over the electrodes for >3 mm long and 150 *μ*m wide [Fig. 1(a)] for controlled measurements. Seven days after platting, electrical activity recording would begin by connecting the MEA chip to an amplifier (MEA 1040, Multichannel Systems) where the temperature was maintained with a TC02 temperature controller (Multichannel Systems). During the recording of spontaneous activity, naturally active cardiomyocytes indicate a field potential signal of the MEAs. These cells have the ability to pace neighboring cells, spontaneously, by sending an impulse that will be followed through the cell pattern. This spontaneous trigger occurs most often unidirectionally although occasionally the impulse will propogate in two directions (left and right). The conduction can travel the length of the pattern (>3 mm), or for shorter segments, if the neighboring cells are not sufficiently stimulated. For analysis of spontaneous conduction, a segment with at least 3 electrodes (600 *μ*m distance) was necessary to ensure an accurate measurement. During the recording of a stimulated activity, an electrode with spontaneous activity was chosen to trigger a controlled impulse using a STG 1002 stimulator (Multichannel Systems). 1 ms bipolar square pulses of amplitude 500–700 mV were applied starting at a lower frequency and escalating in increments (from 0.5 Hz to 2.5 Hz in 0.5 Hz increments). Under these conditions, neighbor cells (in one direction or in both directions) followed the triggered activity and maintained the selected frequency. The conduction traveled the length of the pattern (>3 mm), or shorter segments; however, for an accurate analysis of stimulated activity, a segment with at least 4 electrodes (800 *μ*m distance) was necessary. Recordings would last ≤5 min in total.^31,39,48^ Data collected from the amplifier (MCD file) were converted into ABF files using MC-Data Tool (Multichannel Systems) and further analyzed using Clampfit (Axon Instruments) software. The field potential recorded from the MEA chip shared similarities with the shape to an action potential from a patch-clamp recording (intracellular) and also with the wave form of an EKG (also ECG, electrocardiogram).^49^ Cardiac electrical parameters were analyzed and plotted as beat frequency (hertz), conduction velocity (meter per second) and QT-interval (second), or as a percentage of change compared to the baseline.

### Drug testing

Gap junction modulators are a group of drugs known to promote gap junction coupling and are under study for the prevention of junction disruption during ischemic events.^17,33^ At the cellular level, antiarrhythmic peptides such as ZP123 and ZP1609 promote the gap junction coupling by preserving the phosphorylation of connexin-43. At the cell membrane, these peptides are thought to bind a G-protein coupling receptor inducing PKC (Protein kinase C) activity and, as a consequence, inducing the phosphorylated state of connexin-43. Phosphorylated connexin-43 stabilizes the cell to cell coupling formed by the gap junctions allowing us to buffer the depression of conduction velocity induced by the metabolic stress of an ischemic event.^17,35,44^ Peptides ZP123 [FW (formula weight): 617.55; also known as Rotigaptide and GAP-486]^14,17,33,34,43^ and ZP1609 (FW: 405.33; also known as Danegaptide and WAY-261134)^33,35,38^ were kindly provided by Zealand and were resuspended in ultrapure water to a 100 mM stock solution and stored at −20 °C. An acute dose of 5 *μ*M was selected for both drugs to be studied according to the previously studied range.

### Viability assay

Cardiomyocyte viability was measured after 1 and 7 days of drug incubation using an MTT [3-(4,5-Dimethylthiazol-2-yl)-2,5-Diphenyltetrazolium Bromide] assay.^39^ The results are expressed as the percentage of cell survival relative to the control.

### Immunocytochemistry

Cardiomyocytes (7 DIV) were fixed and stained as described in Ref. 39 at the end point of the drug treatment. The antibody dilutions used were mouse anti-Troponin (Abcam 10214, 1:1000), rabbit anti-Connexin 43 (Sigma-Aldrich, C6219, 1:400), antimouse 568 (Life technologies, A11004, 1:200), and antirabbit 488 (Life Technologies, A11034, 1:200). Nuclei staining was achieved while mounting the cells with the Hard Set Mounting containing DAPI (4′,6-diamidino-2-phenylindole, Vector laboratories, Inc.).

### Ischemia protocol

At 7 DIV, electrical activity from human cardiomyocytes in maintenance medium was recorded to set a baseline, after that medium was replaced with HSL3 Glc-free medium for 48 h of glucose starvation before ischemia. For the human iPSC derived cardiomyocytes, but not for the rat neonatal cardiomyocytes, this 48 h period without glucose, prior to the oxygen depletion, was required to accelerate the ischemia damage (supplementary material 5). The culture medium contained other energy sources (17 amino acids in concentration ranges of 0.02–0.8 mM in the base medium and fatty acids 3.6 *μ*M linoleic acid and 3.6 *μ*M linolenic acid in the medium supplement^50^) available for the cardiomyocytes. Electrical activity was recorded again at 24 and 48 h after medium replacement (8–9 DIV). At 9 DIV, ischemia was induced by reducing the atmospheric oxygen to 0.1%–0.25% for 4 h while in the HSL3 Glc-free medium.^22,23,26,28,34,41,51^ Oxygen reduction (hypoxia) was achieved using a Proox Model 110 (BioSherix) hypoxic chamber, fed with a 5%/95% CO_2_/N_2_ gas mix. The 4 h duration of the ischemic event was selected to induce a change in conduction velocity while preserving the ability of the cardiomyocytes to maintain some electrical function (Figs. 3 and 4, supplementary material 3 and 4). After 4 h of ischemia, oxygen and glucose were reintroduced back into the medium (mimicking a reperfusion) and cardiac electrical activity was measured again at 9 DIV. A full media change was performed before each electrical activity recording of the cardiomyocytes. For the drug testing, the peptide ZP1609 (5 *μ*M) was added 48 h before and during the four hours of ischemia.

### Statistical methods

Values were expressed as mean ± SE of a minimum of three independent experiments. Data were evaluated with unpaired Student's t-Test, run with a two-tail distribution and homo- or heteroscedastic variances when analyzing the overall change between two conditions (i.e., control vs condition). One-way ANOVA (analysis of variance) was used to study changes in one parameter (i.e., time). All statistical analyses were performed with Microsoft Excel, and differences with p-values <0.05 were taken as statistically significant.

## SUPPLEMENTARY MATERIAL

See the supplementary material for spontaneous contraction of human iPSC derived cardiomyocytes after 6 h of ischemia (supplementary material 1); *in vitro* ischemia protocol and effect on human iPSC derived cardiomyocyte morphology (supplementary material 2); ischemia effect on *in vitro* cardiomyocyte stimulated conduction velocity (supplementary material 3); *in vitro* ischemia effect on conduction velocity utilizing patterned human iPSC derived cardiomyocytes (raw data) (supplementary material 4); and *in vitro* ischemia protocol optimization steps for rat compared to human cardiomyocyte cells (supplementary material 5).
